# Renal Denervation for Uncontrolled Hypertension: A Measurement-First, Program-Based Approach

**DOI:** 10.3390/jcm15072648

**Published:** 2026-03-31

**Authors:** Lukasz Szarpak, Burak Katipoglu, Milosz J. Jaguszewski, Andrea Baier, Jacek Kubica, Maciej Maslyk, Michal Pruc, Karol Momot, Basar Cander, Queran Lin

**Affiliations:** 1 Institute of Medical Sciences, Collegium Medicum, The John Paul II Catholic University of Lublin, 20-708 Lublin, Poland; 2 Henry JN Taub Department of Emergency Medicine, Baylor College of Medicine, Houston, TX 77030, USA; 3 Department of Emergency Medicine, Ministry of Health Ankara Etlik City Hospital, Ankara 06170, Türkiye; 4 1st Department of Cardiology, Medical University of Gdansk, 80-214 Gdansk, Poland; 5 Institute of Biological Sciences, Collegium Medicum, The John Paul II Catholic University of Lublin, 20-708 Lublin, Poland; 6 Ludwik Rydygier Collegium Medicum in Bydgoszcz, Nicolaus Copernicus University in Torun, 85-094 Bydgoszcz, Poland; jkubica@cm.umk.pl; 7 3rd Department of Internal Diseases and Cardiology, Międzylesie Specialist Hospital in Warsaw, Medical University of Warsaw, 04-749 Warsaw, Poland; 8 Laboratory of Centre for Preclinical Research, Department of Experimental and Clinical Physiology, Medical University of Warsaw, 02-097 Warsaw, Poland; 9 Department of Emergency Medicine, Bezmialem Vakif University, Fatih, Istanbul 34093, Türkiye; 10 Department of Primary Care and Public Health, School of Public Health, Faculty of Medicine, Imperial College, London W12 OBZ, UK; queran.lin18@alumni.imperial.ac.uk

**Keywords:** ambulatory blood pressure monitoring, home blood pressure monitoring, medication adherence, patient selection, pseudoresistance, renal denervation, resistant hypertension, uncontrolled hypertension

## Abstract

**Background/Objectives**: Renal denervation (RDN) has re-emerged as an adjunctive treatment option for patients with uncontrolled or resistant hypertension, with contemporary sham-controlled trials showing a modest but reproducible reduction in out-of-office blood pressure. However, in routine practice, apparent treatment resistance often reflects pseudoresistance caused by the white-coat effect, poor measurement quality, therapeutic inertia, or nonadherence. This review aimed to summarize the contemporary evidence on renal denervation in uncontrolled or resistant hypertension and to propose a pragmatic, measurement-first framework for patient selection, integration into routine care, and a structured post-procedural response assessment. **Methods**: This article is a narrative, implementation-focused review. A structured search of PubMed, Embase, Cochrane CENTRAL, and Web of Science was performed from database inception through January 2026. We prioritized the randomized sham-controlled RDN trials, major meta-analyses, guidelines, consensus documents, and studies addressing ABPM, HBPM, medication adherence, and telemonitoring. **Results**: The contemporary sham-controlled trials support RDN as an adjunctive option with a modest blood pressure-lowering effect, which is best assessed by out-of-office measurements. The placebo-adjusted reductions in ambulatory systolic blood pressure were generally in the 4–6 mmHg range. Appropriate use requires the confirmation of sustained uncontrolled hypertension, the exclusion of pseudoresistance, the optimization of treatment, and an adherence assessment. We identified three phenotypes most likely to benefit and proposed a three-axis framework for a response assessment at 3 and 6 months. **Conclusions**: RDN should be viewed not as a substitute for antihypertensive therapy but as a program-based adjunct for carefully selected patients. The measurement-first care pathway presented here should be interpreted as a pragmatic clinical model intended to operationalize the available trial and guideline evidence in routine care, rather than as a prospectively validated algorithm or formal consensus recommendation.

## 1. Introduction

Hypertension is still the most common and most fixable driver of cardiovascular disease in the general population [1,2]. Yet the control rates remain disappointing. The World Health Organization estimated that 1.4 billion adults aged 30–79 years had hypertension in 2024, and only a little over one in five achieved adequate control [3,4].

What sits behind those numbers is familiar to anyone running a hypertension clinic: an over-reliance on office readings as definitive, underuse of ambulatory or home blood pressure monitoring, slow treatment intensification, and persistent challenges with adherence to prescribed therapy. The European guidance has leaned hard into the out-of-office confirmation and phenotyping precisely because it helps avoid both undertreatment and overdiagnosis [5,6,7].

True resistant hypertension is generally defined as a blood pressure that remains above the target despite treatment with at least three antihypertensive agents of different classes, typically including a renin–angiotensin system blocker, a calcium channel blocker, and a diuretic, prescribed at maximally tolerated or appropriate doses; it also includes patients whose blood pressure is controlled with four or more drugs [8]. Its true prevalence is substantially lower than that of apparent treatment-resistant hypertension once pseudoresistance, poor measurement quality, white-coat effect, and nonadherence are excluded [9,10]. Clinically, true resistant hypertension is important because it identifies a population at particularly high cardiovascular and renal risk, with limited pharmacological headroom and a greater likelihood of requiring structured multidisciplinary management [11]. This is also the most established phenotype in which renal denervation has been evaluated and is most readily considered in practice.

**Figure 1:**
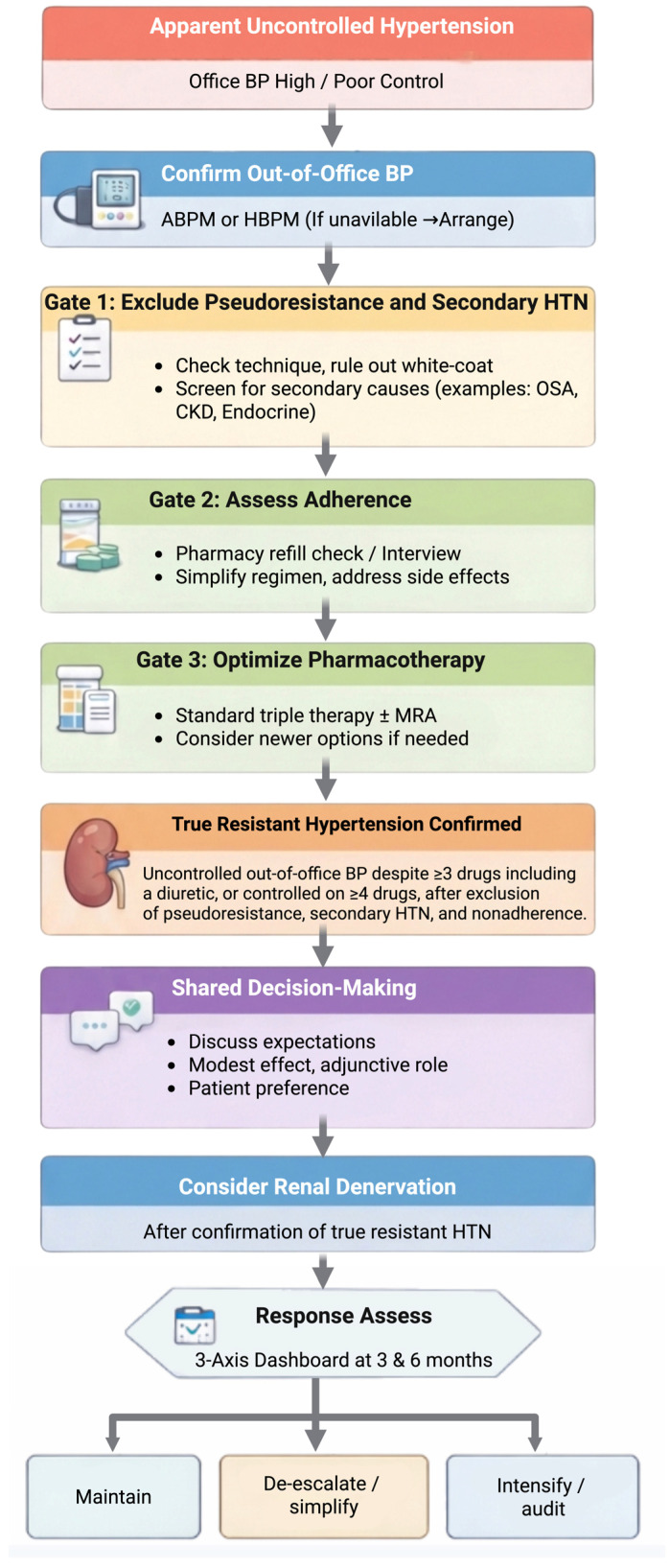
The measurement-first clinical pathway for identifying patients with true resistant hypertension in whom renal denervation may be considered. The pathway emphasizes out-of-office blood pressure (BP) confirmation (preferably ambulatory blood pressure monitoring (ABPM)), exclusion of pseudoresistance and secondary hypertension, adherence assessment, optimization of pharmacotherapy, shared decision-making, and predefined response assessment at 3 and 6 months.

Renal denervation (RDN) has returned to this landscape with better trial design, procedural standardization, and more realistic expectations than a decade ago [12]. The clinically useful question is no longer whether the procedure can lower blood pressure on average but whether it is the right next step for a given patient, with a clear goal, within a care program that can measure and act on outcomes [13,14]. This review is written for clinicians to inform evidence-based decision-making and improve the quality of hypertension care. We summarize this measurement-first, program-based care pathway—from out-of-office confirmation and pseudoresistance exclusion through shared decision-making, renal denervation, and structured follow-up—in Figure 1. What distinguishes this review from guideline summaries and prior general reviews is its explicit focus on clinical implementation. Rather than restating trial results alone, we propose a measurement-first, program-based framework for integrating RDN into routine hypertension care, define practical eligibility gates and clinically relevant phenotypes for patient selection, and introduce a structured three-axis approach to post-procedural response assessment that is based on out-of-office blood pressure, medication exposure/adherence, and data quality. Thus, the manuscript is intended not only as an evidence overview but also as a pragmatic model for translating the contemporary RDN evidence into real-world practice.

## 2. Materials and Methods

This manuscript was designed as a narrative, implementation-focused review intended to inform the integration of renal denervation into contemporary care pathways for uncontrolled hypertension. It was not conceived as a formal systematic review or as a meta-analysis. Nevertheless, to maximize methodological transparency, we used a predefined structured search, explicit eligibility criteria, staged screening, and a prespecified hierarchy for evidence prioritization.

The references for this review were identified through structured searches of PubMed/MEDLINE, Embase, Cochrane CENTRAL, and Web of Science from database inception to 31 January 2026 using database-specific combinations of controlled vocabulary and free-text terms that focused on renal denervation and uncontrolled or resistant hypertension, which were supplemented where appropriate by trial-, device-, and implementation-related terms. Additional relevant publications were identified through the backward and forward citation tracking of pivotal sham-controlled renal denervation trials, major meta-analyses, and contemporary guideline or consensus documents, together with selected regulatory and trial registry sources, which were included when they informed safety, implementation, or longer-term follow-up. This review was conceived as a narrative, implementation-focused synthesis of the literature rather than as a formal systematic review or meta-analysis; therefore, PRISMA-based study selection was not performed. The full search strategies are provided in Supplementary Table S1.

The eligible publications were those directly relevant to the clinical implementation of RDN in adults with uncontrolled or resistant hypertension. The review considered randomized sham-controlled RDN trials, prespecified or adjudicated follow-up analyses, patient-level pooled analyses, systematic reviews and meta-analyses, contemporary guidelines and consensus documents, large registries, and selected implementation studies addressing out-of-office blood pressure assessment, medication adherence, telemonitoring, medication titration, and follow-up pathways. The mechanistic reports, older uncontrolled studies, duplicate reports, conference-only records, and publications without direct relevance to patient selection, measurement strategy, procedural integration, or structured follow-up were not prioritized in the final synthesis when higher-level or more clinically applicable evidence was available.

The screening was performed in two stages. The titles and abstracts were first reviewed for clinical relevance, after which the potentially eligible records underwent full-text assessment. When multiple reports from the same trial program were available, the principal randomized sham-controlled publication served as the anchor report and was supplemented by prespecified follow-up, pooled, or safety analyses where they were clinically informative. The evidence was prioritized hierarchically: first, the adult sham-controlled randomized trials with ambulatory or home blood pressure endpoints; second, the prespecified follow-up and pooled analyses; third, the contemporary hypertension and RDN guidelines or consensus documents; fourth, the systematic reviews and meta-analyses; and fifth, the larger registries and pragmatic implementation reports. Across all categories, the final selection was guided by methodological rigor, recency, and direct applicability to real-world care pathways.

We did not apply a formal study-level risk-of-bias tool because the objective of the manuscript was narrative and practice-oriented rather than quantitative evidence synthesis. Instead, the credibility was judged pragmatically according to the design features that were most relevant to the clinical interpretation, including randomization, sham control, background medication context, preference for out-of-office blood pressure endpoints, follow-up completeness, and direct applicability to contemporary hypertension care.

To further improve transparency, we summarized the search and selection process quantitatively. The structured search identified 1872 records, of which 1317 remained after the removal of duplicates. After title and abstract screening, 198 reports underwent full-text review, and 35 publications were retained for the final narrative synthesis. Because the objective of this work was an implementation-focused narrative review rather than a formal evidence synthesis, PRISMA reporting was not undertaken. An additional limitation is that this work was conducted as a structured narrative review rather than as a formal systematic review with PRISMA-based study selection. Although we used a structured multi-database search, some degree of selection bias in the explicit eligibility criteria, the staged screening, the evidence-prioritization principles, and the full report of the search strategies in Supplementary Table S1 cannot be excluded.

## 3. From “Does It Work?” to “When Does It Make Sense?”

A few millimeters of mercury (mmHg) are important. In large-scale analyses of randomized trials, a 5 mmHg reduction in systolic blood pressure is associated with roughly a 10% lower risk of major cardiovascular events; the larger reductions bring larger average benefits [15,16].

Because the average blood pressure reduction with RDN is modest, it is particularly vulnerable to misclassification and bias if the routine care relies on office readings alone or if medication exposure is uncertain. Apparent resistant hypertension is often pseudoresistance due to white-coat effect, cuff problems, poor technique, drug-induced blood pressure elevation, inadequate diuretic strategy, excess sodium, and (most commonly) nonadherence [5,6,17,18,19].

RDN cannot fix pseudoresistance. If the basics are skipped, invasive therapy may be offered to the wrong patients, and any subsequent “nonresponse” may simply reflect poor measurement, poor data quality, or unstable medication exposure. In current practice, the clinically useful question is therefore utility, not ideology: when does adding a modest, adherence-independent blood pressure component meaningfully increase the probability of sustained target attainment beyond what accurate measurement, adherence support, and guideline-based pharmacotherapy can deliver?

## 4. Measurement-First Hypertension Care

Out-of-office blood pressure measurements best reflect the patient’s usual blood pressure burden. Both the ESH 2023 and the ESC 2024 recommend ambulatory and/or structured home blood pressure monitoring to confirm diagnosis, to identify white-coat and masked phenotypes, and to evaluate apparent treatment resistance [5,6].

Home monitoring is especially useful because it scales and it fits telemedicine. The AHA/American Medical Association (AMA) statement provides a practical blueprint for device validation, patient training, measurement schedules, and interpretation [20]. The European measurement guidance complements this with clear protocols and thresholds across office, home, and ambulatory settings [7].

Masked hypertension and masked uncontrolled hypertension are not academic constructs; they carry an increased risk regardless of whether out-of-office blood pressure is assessed by ambulatory or home methods [21].

For renal denervation, out-of-office data do three jobs at once: they prevent inappropriate referrals based on office-only readings, they provide a stable baseline for judging a modest effect, and they allow us to run post-procedure care as treat-to-target rather than “wait and see”. In practice, a 7-day home series (twice daily, two readings each time, discarding day 1) is a sensible minimum, and ambulatory monitoring remains the preferred way to confirm true resistant hypertension.

For practical implementation, sustained uncontrolled hypertension should be confirmed using out-of-office thresholds rather than office readings alone [22]. A reasonable working definition is a mean 24 h ambulatory blood pressure of at least 130/80 mmHg or a standardized home blood pressure average of at least 135/85 mmHg, which is interpreted together with office values, treatment intensity, and overall cardiovascular risk [8,22]. This is particularly important when considering an intervention with a modest average blood pressure-lowering effect because imprecise phenotyping at baseline can easily lead to an inappropriate referral or a misinterpretation of the post-procedural response.

## 5. Modern Pharmacotherapy and the Persistent Adherence Problem

Most patients can reach target blood pressure with simple, well-executed pharmacotherapy. The ESH 2023 emphasizes early combination therapy, preferential use of single-pill combinations, and a structured approach to resistant hypertension that includes optimizing diuretics and considering a mineralocorticoid receptor antagonist, when tolerated by the patient [5,17].

For the difficult-to-treat patients, the drug pipeline is expanding. Aprocitentan lowered blood pressure in resistant hypertension in a phase three trial [23]. Aldosterone synthase inhibitors, such as baxdrostat and lorundrostat, have demonstrated clinically meaningful blood pressure reductions in uncontrolled and resistant populations [24,25]. RNA interference therapies targeting angiotensinogen (zilebesiran) offer durable blood pressure lowering with dosing intervals measured in months [26,27].

None of this changes the daily reality that nonadherence is common, often hidden, and frequently mistaken for biology. The urine and biochemical screening studies consistently show substantial nonadherence among patients referred to as “resistant”; identifying this can lower blood pressure and improve adherence [18,19].

In this context, RDN may be considered. It should not be used to bypass medication optimization. It may be appropriate when the incremental benefit of further drug intensification is limited by intolerance, regimen complexity, or persistent adherence barriers despite the support received and when a modest, durable reduction is likely to help achieve an agreed blood pressure target.

Although RDN is increasingly positioned alongside newer pharmacological options for resistant or difficult-to-control hypertension, the available comparisons are indirect. Novel agents such as aprocitentan, aldosterone synthase inhibitors, and angiotensinogen-targeting RNA interference therapies may produce substantial blood pressure reductions in selected trial settings, but their effects depend on continued treatment exposure, long-term tolerability, and adherence, and their trial populations differ materially from those enrolled in the contemporary sham-controlled RDN studies. RDN, by contrast, offers a one-time, adherence-independent intervention with modest but reproducible blood pressure lowering across randomized sham-controlled trials. Among non-pharmacological and device-based approaches, RDN currently has the most mature sham-controlled evidence base and the clearest pathway into contemporary clinical guidance, whereas baroreflex activation or amplification and other neuromodulatory strategies remain less established and should presently be regarded as investigational or highly selective options rather than routine comparators in hypertension care [12,24,27,28].

## 6. Renal Denervation: Evidence and Interpretation

The renal sympathetic nerves influence renin secretion, sodium handling, renal hemodynamics, and systemic sympathetic tone. A catheter-based RDN targets these pathways by delivering radiofrequency or ultrasound energy within the renal arteries to ablate peri-arterial nerves [29,30].

The current evidence base stands on sham-controlled trials. In RADIANCE II, ultrasound RDN reduced the daytime ambulatory systolic blood pressure at 2 months when antihypertensive drugs were withdrawn compared with sham, which did not [31]. In RADIANCE-HTN TRIO, a population with resistant hypertension that was treated with a standardized triple single-pill combination, ultrasound RDN again lowered the ambulatory blood pressure versus sham, which did not [32]. For radiofrequency renal denervation, SPYRAL HTN-OFF MED Pivotal demonstrated superiority over sham in lowering 24 h ambulatory systolic blood pressure at 3 months without medications [33]. The contemporary evidence base for renal denervation is grounded in randomized sham-controlled trials using ambulatory endpoints. The key core trials and their placebo-adjusted systolic BP effects are summarized in Table 1.

The trials conducted on background drug therapy illustrate an important clinical point: medication changes and variability in adherence can blunt the observable between-group difference, even when the procedure is biologically active. In SPYRAL HTN-ON MED (on background therapy), the baseline-adjusted between-group difference in the change in 24 h ambulatory systolic BP at 6 months was −1.9 mmHg (95% CI, −4.4 to 0.5; *p* = 0.12). Despite a neutral primary endpoint (which may have been influenced by medication intensification in the control arm), several secondary analyses favored RDN, including a greater reduction in office systolic BP (adjusted treatment difference, −4.9 mmHg; *p* = 0.0015) and signals in nocturnal BP and win ratio analyses [34,35].

The systematic reviews and meta-analyses of sham-controlled trials show a modest but consistent reduction in ambulatory and office blood pressure with catheter-based renal denervation, with no signal for excess major adverse events across the contemporary devices [36]. The patient-level pooled analyses from the RADIANCE program suggest that the blood pressure-lowering effect of ultrasound RDN is maintained at 6 months in the context of protocolized medication escalation [37].

In parallel, RDN has moved from investigational to a clinically adopted procedure in selected jurisdictions. In the United States, the Food and Drug Administration approved both an ultrasound-based system (Paradise) and a radiofrequency system (Symplicity Spyral) in November 2023 as adjunctive treatments to reduce blood pressure in patients whose blood pressure is not adequately controlled with lifestyle modification and antihypertensive medications [38,39].

The safety of the procedures is part of the conversation. Renal artery adverse events appear uncommon, with a low annual rate of renal artery stent implantation reported in a meta-analysis [40]. The registries suggest that there are sustained blood pressure reductions with acceptable renal safety profiles, while acknowledging the limits of observational data [41].

Finally, the field has learned from failure. SYMPLICITY HTN-3 showed how procedural variability and changes in the background therapy can overwhelm a signal, and why the contemporary trials are designed with a tighter control and a better procedural technique [42].

The guidance documents now place RDN as an adjunctive therapy for selected patients and stress the need for accurate measurement, optimization of lifestyle and drugs, and shared decision-making [5,6,12,43].

Although the current randomized evidence for RDN is strongest in the short- to mid-term and primarily addresses blood pressure reduction rather than hard clinical outcomes, the available longer-term follow-up data suggest that the blood pressure-lowering effect may persist beyond the early post-procedural period in selected cohorts [22]. However, this should be interpreted cautiously. As the follow-up extends, the evidentiary base becomes increasingly dependent on the extension studies and registry data, where changes in background pharmacotherapy, medication adherence, and co-interventions may substantially influence the observed trajectories [20]. Thus, the current data support the possibility of a durable blood pressure benefit, but they do not yet establish durable reductions in cardiovascular events, kidney outcomes, or mortality. Similarly, although longer-term observational data suggest generally acceptable renal safety and kidney function trajectories after RDN, the long-term renal outcome benefit remains insufficiently defined and warrants a prospective evaluation.

## 7. Realistic Effect Size: What to Expect and How to Communicate It

In contemporary sham-controlled trials, baseline-adjusted between-group differences in ambulatory systolic BP (daytime or 24 h, depending on the protocol) are typically in the range of −4 to −6 mmHg at 2–6 months (e.g., −6.3 mmHg in RADIANCE II; −4.5 mmHg in RADIANCE-HTN TRIO; −3.9 mmHg in SPYRAL HTN-OFF MED Pivotal) [36].

Two points matter for clinicians: first, the small average effects can still be worthwhile in high-risk patients because BP reductions translate into risk reduction over time [15,16]. Second, the response varies. Some of the patients do substantially better than average, and other patients barely move. That variability is why RDN must be offered as a probability enhancer, not a guarantee, and why the post-procedure plan must include a predefined response assessment and a clear next step.

When we counsel patients, it helps to put the expected change into a familiar frame: a few mmHg on ambulatory or home averages can be the difference between “almost controlled” and “controlled” over the years, particularly in patients with prior stroke, chronic kidney disease, or diabetes, where the absolute risk is high. At the same time, we emphasize what the procedure will not do: it will not normalize the pressure in someone who stops all medication, and it will not rescue a measurement strategy that relies on sporadic office readings. This kind of expectation setting reduces disappointment and, importantly, reduces unsafe self-directed medication withdrawal after the procedure.

## 8. Patient Selection: Eligibility Gates and Clinical Phenotypes

The patient selection should be standardized and checklist-driven. A simple pre-procedure checklist protects patients and improves the interpretability of outcomes in routine care. A practical pre-procedure checklist for patient selection is provided in Table 2.

Gate 1: Confirm the sustained uncontrolled blood pressure using ambulatory monitoring or a standardized home series (never office readings alone) [5,6,7,20].

Gate 2: Actively exclude pseudoresistance, including technique errors, white-coat effect, drug-induced elevation, excess sodium or alcohol, and especially nonadherence. Use the refill data, telemonitoring patterns, and, where available, biochemical testing [17,18,19].

Gate 3: Demonstrate that pharmacotherapy has been optimized and simplified according to the guidelines or clearly document why the escalation is limited (intolerance, polypharmacy, and adverse effects) [5,17].

Gate 4: Consider the secondary causes when clinically indicated and confirm the anatomic feasibility per center protocol.

In this framework, RDN most often makes sense in three clinical phenotypes:

(1) True resistant hypertension: the sustained uncontrolled out-of-office blood pressure despite an optimized regimen and documented exposure; here, RDN can add an incremental component without increasing pill burden [32,33,36].
(2) Limited pharmacologic headroom: the confirmed uncontrolled blood pressure where intensification is constrained by adverse effects or polypharmacy; RDN may close a residual gap once the measurement and adherence are secured [6,12].
(3) Persistent adherence barriers in high-risk patients: RDN adds redundancy, but only within a broader program that includes telemonitoring and adherence support [12,44,45].

These practical phenotypes and suggested, measurable 3–6-month goals are summarized in Table 3.

Certain subgroups require additional caution when translating these phenotypes into practice. In patients with chronic kidney disease, candidacy should be judged not only by blood pressure phenotype but also by baseline kidney function, expected contrast burden, potassium handling, and renal artery anatomy [12,46]. In older adults, the chronological age alone should not be used as an exclusion criterion; instead, frailty, orthostatic symptoms, cognitive and functional status, polypharmacy, and likely tolerance of subsequent titration should guide the individualized decisions [47,48]. By contrast, patients with suspected or untreated secondary hypertension should not proceed directly to renal denervation, because the identification and treatment of the underlying cause may substantially alter the blood pressure trajectory and may obviate the need for invasive therapy.

The patients with isolated white-coat hypertension, absent or poor-quality out-of-office data, untreated secondary hypertension, or obvious reversible drivers that are not addressed are unlikely to benefit and should not proceed with the invasive therapy.

For patients with CKD, candidacy should be based not only on the blood pressure phenotype but also on the expected procedural feasibility, renal artery anatomy, baseline kidney function, and whether a modest but durable blood pressure reduction is likely to alter the patient’s near-term clinical trajectory [12].

## 9. Practical Exclusions: Defer Renal Denervation Until These Are Addressed

Because the expected BP-lowering effect of renal denervation is modest, an accurate out-of-office confirmation and an exclusion of pseudoresistance are essential to avoid inappropriate referrals and the misclassification of “nonresponse”. In routine practice, renal denervation should be deferred until the following issues are addressed:

- Isolated white-coat hypertension or uncontrolled office readings without confirmatory ambulatory or structured home data.
- Poor-quality out-of-office measurements (unvalidated device, incorrect cuff size, poor technique, or incomplete series).
- Unassessed or likely nonadherence (no objective medication exposure checks and frequent regimen changes without documentation).
- Reversible contributors that are not yet managed (e.g., NSAIDs/systemic steroids, sympathomimetics, excess sodium or alcohol, licorice, and stimulants).
- Suspected secondary hypertension or untreated obstructive sleep apnea when the clinical features warrant an evaluation.
- When pharmacotherapy is not optimized/simplified per the guidelines’ instruction, or there is no clear documentation of why the escalation is limited.
- The anatomic or renal-function constraints per the local protocol (e.g., unsuitable renal artery anatomy or the inability to undergo required imaging).

In practical terms, the pseudoresistance screen should be explicit. A useful checklist includes substances that raise blood pressure (nonsteroidal anti-inflammatory drugs, systemic corticosteroids, sympathomimetics, some antidepressants, and oral contraceptives) and direct questions about the consumption of licorice, alcohol, and recreational stimulants. Sleep should also be treated as a blood pressure variable: if obstructive sleep apnea is likely, then it deserves an evaluation because effective therapy can reduce systolic pressure by an amount comparable to many add-on interventions. The point is not to delay care indefinitely; it is to avoid offering an invasive, modest-effect procedure when a reversible driver is still in play. When the basics are closed, RDN becomes a rational next step rather than an attractive shortcut.

## 10. Renal Function and Anatomic Considerations: Practical Guardrails

Kidney function deserves special attention because the incremental benefit of RDN is modest. When the estimated glomerular filtration rate is reduced or there is a history of contrast intolerance, the team should weigh whether the procedure will change the patient’s trajectory more than intensified pharmacotherapy and monitoring. Anatomic complexity (multiple accessory arteries or very distal branching) may also reduce the likelihood of the patient being completely treated, which should be discussed transparently during the shared decision-making period. These are not reasons to abandon the procedure in all cases; they are reasons to tighten the selection and to align expectations with what is technically achievable in that individual patient. The patients with advanced chronic kidney disease, borderline renal function, or suspected secondary hypertension require an individualized multidisciplinary review before renal denervation is considered, and in many such cases, invasive treatment should be deferred until the underlying phenotype and expected procedural benefit are clarified [49,50].

The procedural appropriateness also depends on the kidney function and the renal artery anatomy. In routine practice, the patients who create the most trouble are those with borderline feasibility for treatment due to prior renal artery stenting, significant renal artery stenosis, fibromuscular dysplasia, aneurysmal disease, very small-caliber arteries, or complex accessory arteries where complete treatment may be difficult. These situations are not classed as absolutely “never” being able to happen across all the systems and protocols, but they should prompt a higher bar for their expected benefit and should prompt careful multidisciplinary discussion [43].

From a guardrail perspective, centers should adopt a simple pre-procedure checklist: baseline estimated glomerular filtration rate, history of renal vascular disease or intervention, and targeted imaging when clinical suspicion is present or when the feasibility is uncertain [12]. The goal is feasibility and safety, not indiscriminate screening. The safety analyses suggest a low rate of major renal artery events, but even rare events matter when the expected blood pressure benefit is modest [40].

The registry data suggest that the renal function trajectories after RDN are generally acceptable in contemporary cohorts, but long-term renal outcomes remain an important area for monitoring and reporting in real-world programs [41].

In patients with chronic kidney disease, renal denervation should not be viewed solely through the lens of procedural caution. In appropriately selected patients, the available CKD-specific literature suggests that the procedure is feasible and is not associated with a consistent signal of excess renal vascular complications or an accelerated decline in kidney function, while still offering a clinically meaningful blood pressure reduction. However, this more favorable interpretation applies only when the selection is rigorous: truly uncontrolled out-of-office blood pressure should be confirmed, pseudoresistance and untreated secondary causes should be excluded, renal artery anatomy should be suitable, and renal function and contrast-related risk should be assessed individually. The evidence is more limited in advanced CKD, and current data do not yet establish the long-term renal or cardiovascular outcome benefits; therefore, in this population, RDN should be considered a carefully selected adjunctive option rather than a routine pathway.

## 11. Shared Decision-Making: Making Renal Denervation a Therapeutic Contract

The choice to proceed with RDN is a preference-sensitive decision. The average effect is modest, individual responses vary, and hard-outcome trials are not yet available. For that reason, the guidance documents emphasize that shared decision-making should be the standard practice [12,43].

The most effective way to run shared decision-making in clinics is to make it concrete. Start by asking what the patient wants: a lower long-term risk by reaching target blood pressure, fewer tablets, fewer adverse effects, or simply more stable control. Then show the data: ambulatory or home averages, current drug regimen, and evidence of medication exposure. This should be followed by offering alternatives honestly: intensified home monitoring with protocolized titration, optimization of single-pill combinations, treatment of reversible drivers (such as sleep apnea), and newer drug classes when appropriate [25,26,27,28,29,30,31,32,33,44,45].

The clinicians should then discuss RDN as an adjunct: on average, it behaves like adding one effective drug in terms of out-of-office blood pressure measurements, but it does not usually eliminate the need for tablets. Finally, end the conversation with a contract: the metric (ambulatory monitoring if possible), the time points (3 and 6 months), and the next step if the goal is not met.

## 12. Integrating Renal Denervation with Home Monitoring and Medication Optimization

RDN does not compete with telemonitoring; it relies on it. The telemedicine trials and meta-analyses show that structured home monitoring combined with feedback and medication titration improves control compared with the usual care [44,45].

Digital health can either narrow or widen disparities. The evidence suggests that tailored interventions can improve outcomes in underserved populations; therefore, a RDN program should ensure that access to validated devices and follow-up is part of the package, not an optional extra [4,47].

A pragmatic care pathway has two phases. Before the procedure, validate the device, train the patient, obtain a reliable home series and (when feasible) ambulatory monitoring, optimize and simplify pharmacotherapy, and address the reversible drivers. After the procedure, avoid long periods of passive observation, keep medications stable long enough to interpret an early change, then titrate to the target using home monitoring and a clear protocol. Someone must own the post-procedure titration; the ambiguity here is a reliable way to lose any procedure effect in the noise.

A small procedural effect becomes clinically useful only if it is protected from the noise. This requires agreeing up front on a short period of relative medication stability (typically 3–6 weeks) unless the safety protocol dictates otherwise, followed by protocolized titration that is guided by home averages. A workable protocol uses simple thresholds that trigger contact (for example, a weekly mean above target by ≥5 mmHg, or symptoms suggesting hypotension), with a clear ownership of decisions. If home averages fall below the target and the patient is symptomatic, de-escalation should be deliberate and documented, as otherwise subsequent “success” or “failure” cannot be interpreted. The programs that do this well feel less like an interventional event and more like chronic disease management with an additional tool. The practical pathway outlined below should be interpreted as an implementation-oriented, expert-informed model derived from available trial evidence, guideline recommendations, and clinical follow-up principles, rather than as a formally validated post-RDN management algorithm. Within this pathway, the “three-axis dashboard” refers to a structured response-assessment framework that integrates out-of-office blood pressure, medication exposure/adherence, and data quality, while considering the safety of the patient as a parallel domain.

### Assessing the Effect at 3–6 Months: A Practical Response Framework

**Figure 2:**
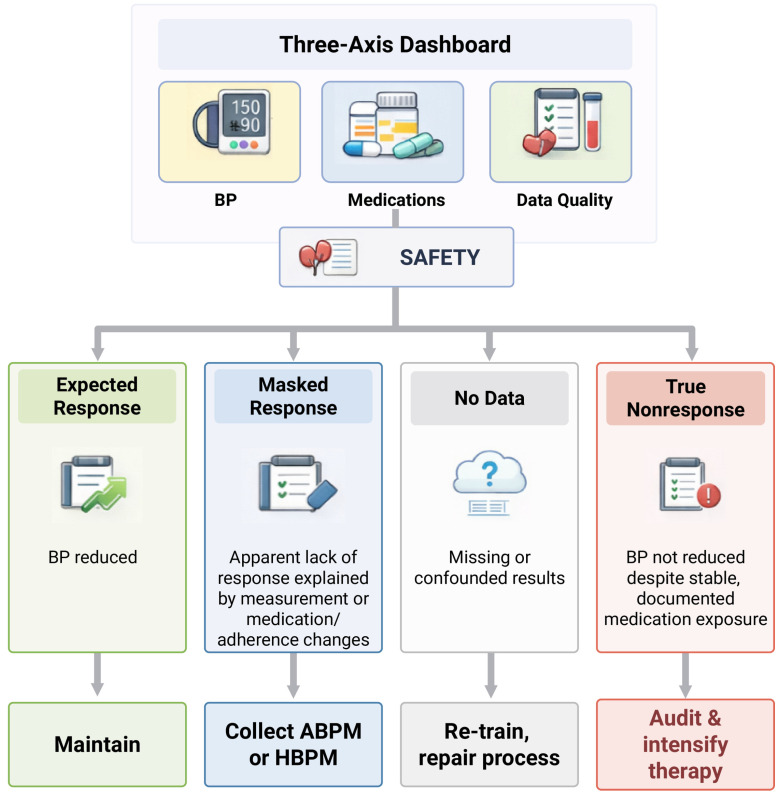
The proposed expert-informed, implementation-focused decision framework for the response assessment after renal denervation at 3 and 6 months using a three-axis dashboard (out-of-office blood pressure (BP), medication exposure/adherence, and data quality), with the safety of the patient assessed as a parallel domain. The framework is intended as a pragmatic tool to support the structured follow-up in routine care and has not been prospectively validated as a formal clinical algorithm.

If you do not define the response up front, you will argue about it later. To operationalize the response assessment and reduce the misclassification of “nonresponse”, we propose a decision-flow dashboard that is evaluated at 3 and 6 months (Figure 2). If the response is not defined prospectively, it is difficult to interpret whether post-procedural blood pressure change reflects the biological effect of RDN, concurrent medication changes, or limitations in the measurement quality. To support a structured follow-up and reduce the misclassification of apparent “nonresponse” in routine practice, we propose an expert-informed, implementation-focused decision framework that is evaluated at 3 and 6 months (Figure 2 and Table 4). This three-axis dashboard is intended to operationalize the available evidence from sham-controlled trials, contemporary guidance, and practical hypertension care principles; however, it should be interpreted as a conceptual clinical tool rather than as a prospectively validated algorithm. At 3 and 6 months, the dashboard evaluates:

- Out-of-office blood pressure: ambulatory monitoring is preferred; otherwise use standardized home averages [7,20].
- Medication exposure and adherence: document medication changes, use refill and telemonitoring indicators, and consider biochemical testing in selected resistant cases [17,18,19].
- Data quality: a validated device, the correct technique, and a complete measurement series.

At the follow-up, classify patients into four scenarios: expected response; masked response (explained by medication drift, adherence changes, or confounders); no data; and true nonresponse. The key quality marker is the third category: no data is not the same as no response. Incomplete ambulatory or home monitoring should trigger the process of repair and repeat measurement, not a judgment of inefficacy.

A pragmatic threshold for hemodynamic response is a 5 mmHg or greater reduction in a 24 h ambulatory systolic blood pressure, which aligns with the typical placebo-adjusted effect size observed in contemporary sham-controlled trials and pooled analyses, which are interpreted alongside the medication stability or a planned, documented de-escalation [36].

When true nonresponse is suspected, the first move is an audit: verify the measurements, reassess the adherence, ensure a guideline-based optimization (including diuretic strategy and a mineralocorticoid receptor antagonist when appropriate), revisit secondary causes and drug-induced contributors, and review procedural quality within the center’s governance framework [5,6,12].

A typical real-world pitfall is as follows: a patient returns at 3 months with one excellent office reading, reports dizziness, and has quietly halved the diuretic dose. Without home or ambulatory averages, the team declares “great response”, and the patient later rebounds with uncontrolled home readings. The converse is just as common and can be seen as follows: the office pressure remains high, but the home averages are meaningfully lower, and the apparent “failure” is a white-coat phenotype that was never addressed. A dashboard approach prevents both errors by forcing us to look at the out-of-office data, the medication exposure, and the data quality before we judge the procedure.

## 13. Implementation, Quality, and Equity

The major risk of RDN is not the catheter; it is misplacement in the care pathway [12,48]. If we treat pseudoresistance or offer the procedure without a monitoring infrastructure, we will waste resources and disappoint patients.

A credible program should track a small set of quality indicators: the proportion of referrals with confirmed uncontrolled out-of-office blood pressure; the documentation of adherence assessment; the time to medication optimization and titration; the proportion reaching the agreed target at 6 months; and the standardized safety reporting [12,40,41,43] (vascular complications and renal function).

A practical way to prevent procedure-led decision-making is to formalize a pre-procedure checkpoint signed off by the longitudinal care team. This checkpoint should confirm a sustained out-of-office hypertension on a validated device, a documented optimization attempt with simplified dosing (preferably single-pill combinations), a structured adherence assessment, and a patient-defined goal that cannot reasonably be met by monitoring and titration alone [10]. Where telemonitoring workflows exist, the checkpoint becomes the bridge between the interventional episode and the chronic disease management: it specifies who owns the post-procedure titration, what thresholds trigger contact, and how the adverse effects and hypotension are handled [49,50,51].

Equity is not optional. The World Health Organization has emphasized that validated devices and reliable access to antihypertensive medicines are prerequisites for population control [4]. A RDN pathway that assumes digital access without providing the support risks becomes a preferential service for those who have already been well served. The evidence base for digital health suggests that a tailored implementation can improve the outcomes in underserved groups; the programs should be designed for that from day one [47].

## 14. Future Directions

The most important evidence gap remains the lack of adequately powered randomized trials for hard clinical outcomes. Additional priorities include the validated predictors of response, the sequencing studies comparing RDN with emerging drug classes and intensive telemonitoring, and the pragmatic implementation of trials that measure effectiveness, cost-effectiveness, and equity.

From a clinical implementation standpoint, the most decisive next step would be pragmatically randomized or registry-based studies with a standardized out-of-office BP measurement that reports (1) the target attainment and time-in-range, (2) the medication burden and tolerability (including changes in pill count and dose intensity), (3) the adherence stability (refill data, digital monitoring, or biochemical verification in selected cohorts), and (4) the renal/vascular safety regarding prespecified imaging and kidney function trajectories. A follow-up of at least 2–3 years would better capture the durability, treatment sequencing, and clinically meaningful downstream outcomes.

Until those data arrive, the best service we can do for patients is discipline: careful selection, honest expectation-setting, and follow-up plans that treat blood pressure control as a process rather than a single event.

## 15. Limitations and Evidence Gaps

The current RDN evidence is strongest for lowering blood pressure, where sham-controlled trials show consistent, modest reductions; however, the field still lacks adequately powered randomized trials that demonstrate the benefits on hard clinical endpoints such as cardiovascular events, kidney outcomes, or mortality. The study populations remain heterogeneous across the severity of hypertension, the baseline risk, the background pharmacotherapy (ON-med vs. OFF-med designs), and the procedural approaches, which limits the generalizability and makes the effect sizes difficult to compare. In addition, the changes in concomitant medications and variable adherence over follow-up can materially confound the observed blood pressure trajectories, particularly in routine practice where medication intensification and lifestyle changes frequently occur in parallel. The outcome ascertainment also varies (office vs. ambulatory vs. home measurements), and the follow-up is often limited to months, leaving the durability beyond the mid-term less precisely defined. To mitigate these limitations in clinical implementation, our proposed program standardizes the out-of-office blood pressure assessment, requires a medication stabilization period with a structured adherence evaluation, and mandates the prospective documentation of all therapy changes. Finally, the predefined follow-up time points and endpoints (e.g., 24 h ambulatory systolic blood pressure at 3 and 6 months) are coupled to an explicit nonresponse pathway, ensuring that suboptimal control triggers systematic reassessment rather than therapeutic drift. An additional limitation of this review is that the proposed measurement-first care pathway and three-axis response dashboard are not themselves derived from prospective validation studies and should not be interpreted as formal consensus recommendations. Rather, they represent an expert-informed, implementation-oriented synthesis that is intended to help translate trial evidence, guideline principles, and routine hypertension care requirements into a structured clinical workflow. Their clinical utility and reproducibility should ideally be examined in future prospective implementation studies.

## 16. Key Take-Home Messages

- Confirm the sustained uncontrolled blood pressure with ABPM and/or structured HBPM before referral.
- Systematically exclude pseudoresistance, with particular attention to the measurement technique and nonadherence.
- Optimize and simplify pharmacotherapy and address the reversible drivers (e.g., excess sodium, drug-induced hypertension, sleep apnea); RDN is adjunctive, not a shortcut.
- Consider the structured response assessment at 3 and 6 months using predefined out-of-office targets together with a pragmatic three-axis dashboard (blood pressure, data quality, and medication trajectory) to reduce the misclassification in routine care.
- Interpret the durability against the medication trajectory and out-of-office averages; a key clinical benefit may be a reduced need for repeated intensification to maintain the target control.

## 17. Conclusions

RDN is not a substitute for evidence-based antihypertensive pharmacotherapy. It is an adjunctive option with a modest but reproducible effect on the out-of-office blood pressure. Whether that effect is worth pursuing depends on selecting the right patient and embedding the procedure in a care pathway that captures clinically relevant parameters.

A pragmatic, measurement-first, program-based approach may help integrate RDN into routine care and confirm the sustained uncontrolled out-of-office blood pressure, exclude pseudoresistance and nonadherence, optimize and simplify pharmacotherapy, integrate RDN into telemonitoring, and reassess at 3 and 6 months with explicit next-step rules. This pathway should be interpreted as an expert-informed implementation model rather than as a validated algorithm, but it may help frame the use of RDN in patients with true resistant hypertension, limited pharmacologic headroom, or persistent adherence barriers in high-risk settings.

The principal contribution of this review is not a new efficacy claim for RDN but a pragmatic framework for translating contemporary trial and guideline evidence into patient selection, longitudinal care, and structured response assessments in routine practice. At present, the most defensible interpretation of the long-term evidence is that RDN may provide a durable blood pressure-lowering effect in selected patients, but a durable cardiovascular or renal outcome benefit has not yet been proven.

## Figures and Tables

**Table 1:** The sham-controlled randomized trials of renal denervation: selected core evidence.

Trial	Technology	Population/Background Therapy	Primary SBP Endpoint (ABPM)	Primary Endpoint Time Point	Sham-Adjusted Between-Group Difference in Change in SBP
RADIANCE II	Ultrasound	Hypertension; antihypertensive medications withdrawn	Daytime ambulatory SBP (ABPM)	2 months	−6.3 mmHg (95% CI, −9.3 to −3.2; *p* < 0.001)
RADIANCE-HTN TRIO	Ultrasound	Resistant hypertension; standardized triple single-pill combination (run-in)	Daytime ambulatory SBP (ABPM)	2 months	−4.5 mmHg (95% CI, −8.5 to −0.3; adjusted *p* = 0.022)
SPYRAL HTN-OFF MED Pivotal	Radiofrequency	Hypertension; antihypertensive medications withdrawn	24 h ambulatory SBP (ABPM)	3 months	−3.9 mmHg (Bayesian 95% CrI, −6.2 to −1.6)
SPYRAL HTN-ON MED	Radiofrequency	Hypertension on background antihypertensive medications	24 h ambulatory SBP (ABPM)	6 months	−1.9 mmHg (95% CI, −4.4 to 0.5; *p* = 0.12)

Abbreviations: ABPM, ambulatory blood pressure monitoring; SBP, systolic blood pressure; CI, confidence interval; and CrI, credible interval. The between-group differences are sham-adjusted in the change from the baseline at the primary endpoint time point.

**Table 2:** The patient selection checklist for renal denervation (RDN).

The confirmed uncontrolled hypertension on the out-of-office measurement (24 h ABPM preferred; HBPM acceptable), with the white-coat effect excluded. Optimized guideline-directed pharmacotherapy (including a diuretic where indicated) with an attempt at a mineralocorticoid receptor antagonist unless contraindicated or not tolerated. The structured adherence assessment is completed and documented (e.g., interview plus pharmacy refill review; consider drug level testing where available). The secondary hypertension evaluation is performed when clinically indicated (e.g., primary aldosteronism, renal artery stenosis, obstructive sleep apnea). The medication regimen is stabilized for an agreed period (e.g., 4–6 weeks) before the baseline ABPM/HBPM and before the procedure, unless safety protocols dictate changes. The suitable renal anatomy is confirmed by imaging, and procedural eligibility is verified; the renal function within the center’s accepted threshold and risks are reviewed. The shared decision-making is completed, including realistic expectations (adjunct therapy, modest average effect, and need for an ongoing follow-up). The center capacity for standardized follow-up (ABPM/HBPM schedule, medication reconciliation, and nonresponse pathway) is confirmed.

**Table 3:** The practical phenotypes for considering renal denervation and the suggested, measurable goals.

Phenotype	When RDN May Add Value (Core Requirement)	Primary Goal	Must-Have Prerequisites Before RDN	Measurable Success at 3–6 Months (Out-of-Office)
True resistant hypertension	Uncontrolled out-of-office BP despite the optimized regimen and the documented medication exposure.	Close the residual gap to the target without increasing the pill burden.	Confirm the sustained uncontrolled BP by ABPM (preferred) or standardized HBPM Exclude pseudoresistance and reversible contributors Optimize the diuretic strategy; add MRA when tolerated Document the stable regimen and exposure (refill/telemonitoring ± biochemical testing in selected cases)	Target achieved and/or ≥5 mmHg reduction in 24 h (or daytime) ambulatory SBP versus baseline on a stable, documented regimen.
Limited pharmacologic headroom	Confirmed uncontrolled out-of-office BP with intolerance, adverse effects, or polypharmacy limiting escalation.	Improve control without adding adverse effects or complexity.	Simplify the regimen (single-pill combinations where possible) Address the reversible drivers; consider secondary causes when indicated Document the constraints to further escalation (e.g., intolerance, hypotension, electrolyte issues)	Target achieved with unchanged or reduced medication load (dose intensity/pill burden) and without new treatment-limiting adverse effects.
Persistent adherence barriers (high risk)	Confirmed uncontrolled out-of-office BP plus repeated nonadherence despite support, with high baseline cardiovascular risk.	Add an adherence-independent BP-lowering component as redundancy within a monitored program.	The telemonitoring workflow and adherence-support program are put in place Explicit shared decision-making contract (expectations; no self-directed medication withdrawal) The objective exposure checks are feasible (refill/telemonitoring; biochemical testing in selected cases)	Improved out-of-office BP mean (ABPM/HBPM) and reduced time above target thresholds, with documented engagement in monitoring and medication plan.

Abbreviations: ABPM, ambulatory blood pressure monitoring; HBPM, home blood pressure monitoring; BP, blood pressure; SBP, systolic blood pressure; MRA, mineralocorticoid receptor antagonist; and mmHg, millimeters of mercury.

**Table 4:** The proposed expert-informed three-axis dashboard for response assessment after renal denervation at 3 and 6 months, with safety as a parallel domain.

Domain	Preferred Metric	What to Document (Minimum Dataset)	Interpretation	Next Step If Unfavorable
Out-of-office BP (hemodynamic response)	ABPM (preferred) or standardized HBPM averages	Baseline vs. follow-up comparability (same platform, same schedule) Baseline and follow-up mean SBP (and DBP, if available) Any intercurrent illness or major lifestyle change	Judge BP change only on the same measurement platform; avoid office-only interpretation. If medications changed, interpret alongside exposure documentation.	Repair measurement process (training/device) and repeat ABPM/HBPM; then treat-to-target titration.
Medication exposure and adherence	Current regimen + objective exposure indicators (refill/telemonitoring; biochemical testing in selected resistant cases)	Complete medication list (drug, dose, timing) Changes since baseline (intensification/de-escalation; rationale) Objective exposure signal (refill gaps, telemonitoring patterns, biochemical test if used)	Distinguish true nonresponse from medication drift or nonadherence. A stable, documented regimen is required to label ‘true nonresponse’.	Audit adherence and barriers; simplify regimen (single-pill combinations); protocolized titration; consider escalation (e.g., optimized diuretics/MRA when tolerated).
Data quality (measurement integrity)	Validated device and complete HBPM/ABPM dataset	Device validation status and correct cuff size Technique (rest, posture, timing) and completeness (e.g., 7-day HBPM series) Missingness, artifacts, or inconsistent readings	Treat ‘no data’ separately from ‘no response’. Poor-quality or incomplete datasets preclude response classification.	Re-train, replace/validate device, repeat monitoring; avoid premature conclusions or medication changes driven by office readings alone.
Safety (parallel domain)	Renal function and vascular events	Serum creatinine/eGFR at baseline and follow-up Adverse events (vascular access, renal artery events) Any imaging performed per local protocol	Provides context for medication adjustments and informs whether further titration is appropriate.	Adjust therapy per protocol; evaluate adverse events; consider imaging/nephrology/vascular review as indicated.

Note: This dashboard is presented as a pragmatic, implementation-oriented framework to support the interpretation of follow-up data in routine care. It should not be interpreted as a formally validated algorithm or as a substitute for individualized clinical judgment. Abbreviations: ABPM, ambulatory blood pressure monitoring; HBPM, home blood pressure monitoring; BP, blood pressure; SBP, systolic blood pressure; DBP, diastolic blood pressure; eGFR, estimated glomerular filtration rate; and MRA, mineralocorticoid receptor antagonist.

## Data Availability

No new data were created or analyzed in this study. Data sharing is not applicable to this article.

## Supplementary Materials

The following supporting information can be downloaded at https://www.mdpi.com/article/10.3390/jcm15072648/s1, Table S1: Electronic search strategy and additional sources.

## Abbreviations

The following abbreviations are used in this manuscript:

ABPM	Ambulatory blood pressure monitoring
AHA	American Heart Association
AMA	American Medical Association
BP	Blood pressure
CI	Confidence interval
Crl	Credible interval
DBP	Diastolic blood pressure
eGFR	Estimated glomerular filtration rate
ESC	European Society of Cardiology
ESH	European Society of Hypertension
FDA	Food and Drug Administration
HBPM	Home blood pressure monitoring
mmHg	Millimeters of mercury
MRA	Mineralocorticoid receptor antagonist
NSAIDs	Nonsteroidal anti-inflammatory drugs
OFF-med	Off medication
ON-med	On medication
RDN	Renal denervation
SBP	Systolic blood pressure
