# Supplementary material for: Renal Denervation for Uncontrolled Hypertension: A Measurement-First, Program-Based Approach
Source: J Clin Med. 2026 Mar 31;15(7):2648. doi: 10.3390/jcm15072648 (PMC13073221; doi:10.3390/jcm15072648)
Supplement: Supplementary file 1 [file jcm-15-02648-s001.zip › jcm-4220018-supplementary.pdf]

Table S1. Electronic search strategy and additional sources

Database / platform	Search string (verbatim)	Limits / filters
MEDLINE (PubMed)	("renal denervation"[MeSH Terms] OR "renal denervation"[Title/Abstract] OR "renal sympathetic denervation"[Title/Abstract] OR "catheter-based renal denervation"[Title/Abstract] OR "radiofrequency renal denervation"[Title/Abstract] OR "ultrasound renal denervation"[Title/Abstract] OR RDN[Title/Abstract]) AND (hypertension[MeSH Terms] OR hypertension[Title/Abstract] OR "resistant hypertension"[Title/Abstract] OR "uncontrolled hypertension"[Title/Abstract] OR "refractory hypertension"[Title/Abstract]) AND (randomized[Title/Abstract] OR randomised[Title/Abstract] OR "randomized controlled trial"[Publication Type] OR sham[Title/Abstract] OR "sham-controlled"[Title/Abstract])	Humans; Adults (>=18 y) where available; English-language; RCT/clinical trial prioritised at screening
Embase (Elsevier)	('renal denervation'/exp OR 'renal denervation':ti,ab OR 'renal sympathetic denervation':ti,ab OR 'catheter based renal denervation':ti,ab OR 'radiofrequency renal denervation':ti,ab OR 'ultrasound renal denervation':ti,ab OR rdn:ti,ab) AND ('hypertension'/exp OR hypertension:ti,ab OR 'resistant hypertension':ti,ab OR 'refractory	Humans; Adults; English-language; report if conference abstracts excluded

	hypertension':ti,ab OR 'uncontrolled hypertension':ti,ab) AND ('randomized controlled trial'/exp OR random*:ti,ab OR sham:ti,ab OR 'sham controlled':ti,ab)	
Cochrane CENTRAL	("renal denervation" OR "renal sympathetic denervation" OR "catheter-based renal denervation" OR "radiofrequency renal denervation" OR "ultrasound renal denervation" OR RDN) AND (hypertension OR "resistant hypertension" OR "refractory hypertension" OR "uncontrolled hypertension")	Trials database; no language limit at search stage (recommended); screened to eligible full texts
Web of Science Core Collection	TS=((renal NEAR/2 denervation) OR ("renal sympathetic denervation") OR ("catheter-based renal denervation") OR ("radiofrequency renal denervation") OR ("ultrasound renal denervation") OR RDN) AND TS=(hypertension OR "resistant hypertension" OR "refractory hypertension" OR "uncontrolled hypertension") AND TS=(random* OR sham OR "sham-controlled")	English-language; document types reported (Article/Review/Proceedings Paper as applied)

Additional sources (hand-search)

Backward and forward citation tracking of included sham-controlled RDN trials and key meta-analyses; targeted searches of major guideline/consensus documents and society statements (eg, ESH, ESC, AHA/ACC, ISH); regulatory and device safety/approval documentation

(eg, FDA device approvals); trial registries (ClinicalTrials.gov and WHO ICTRP) for ongoing/completed sham-controlled RDN trials and long-term follow-up; and relevant national/center registries where available.
